# Phenotypic and genotypic characterization of *Neisseria gonorrhoeae* isolates from Yaoundé, Cameroon, 2019 to 2020

**DOI:** 10.1099/mgen.0.001091

**Published:** 2023-08-17

**Authors:** Carolle Yanique Tayimetha, Longdoh Anna Njunda, Blaise Akenji, Raspail Carrel Founou, Vitalis Feteh, Denis Zofou, Anicet Chafa, Yannick Oyono, Bienvenu Etogo, Dorine Tseuko, Marie Christine Fonkoua, Odile B. Harrison

**Affiliations:** ^1^​ Faculty of Health Sciences of the University of Buea, Buea, Cameroon; ^2^​ National Public Health Laboratory, Yaounde, Cameroon; ^3^​ Department of Microbiology, Haematology and Immunology of University of Dschang, Dschang, Cameroon; ^4^​ Medical Bacteriology Laboratory of University Hospital Center, Yaoundé, Cameroon; ^5^​ Centre Pasteur du Cameroon, Yaoundé, Cameroon; ^6^​ Cameroonian Society of Microbiology, Yaoundé, Cameroon; ^7^​ Department of Biology, University of Oxford, Oxford, UK; ^8^​ Nuffield Department of Population Health, University of Oxford, Oxford, UK

**Keywords:** gonorrhoea, antimicrobial resistance, genomics, Cameroon, Africa, cgMLST

## Abstract

This study investigated antimicrobial resistance (AMR) phenotypes and genotypes exhibited by *Neisseria gonorrhoeae* from Yaoundé, Cameroon. AMR to tetracycline, penicillin and ciprofloxacin was observed although none of the isolates had reduced susceptibility to azithromycin, cefixime or ceftriaxone. Whole genome sequence (WGS) data were obtained and, using a threshold of 300 or fewer locus differences in the *N. gonorrhoeae* core gene multilocus sequence typing (cgMLST) scheme, four distinct core genome lineages were identified. Publicly available WGS data from 1355 gonococci belonging to these four lineages were retrieved from the PubMLST database, allowing the Cameroonian isolates to be examined in the context of existing data and compared with related gonococci. Examination of AMR genotypes in this dataset found an association between the core genome and AMR with, for example, isolates belonging to the core genome group, Ng_cgc_300 : 21, possessing GyrA and ParC alleles with amino acid substitutions conferring high-level resistance to ciprofloxacin while lineages Ng_cgc_300 : 41 and Ng_cgc_300 : 243 were predicted to be susceptible to several antimicrobials. A core genome lineage, Ng_cgc_300 : 498, was observed which largely consisted of gonococci originating from Africa. Analyses from this study demonstrate the advantages of using the *N. gonorrhoeae* cgMLST scheme to find related gonococci to carry out genomic analyses that enhance our understanding of the population biology of this important pathogen.

## Data Summary

Accession numbers for all sequence reads included in the study are available as supplementary material, available in the online version of this article, project accession number PRJEB58547, including any metadata used and antimicrobial resistance genotypic information. In addition, all whole genome sequence assemblies are publicly available on the pubMLST.org/neisseria database (https://pubmlst.org/neisseria) with isolate id numbers provided in the supplementary material.

**Impact Statement**

Along with *Chlamydia trachomatis, Neisseria gonorrhoeae* causes one of the most widespread bacterial sexually transmitted infections (STIs) worldwide and is a global public health concern due to increasing resistance to multiple antimicrobials. Africa, in particular, suffers the greatest burden of STIs, but limited quality-assured African antimicrobial resistance (AMR) data, particularly from Central and Western Africa, are available causing concern for the capacity to limit AMR in this region and highlighting an urgent need to develop the infrastructure needed to enhance surveillance. In Cameroon, ciprofloxacin was the first-line treatment for gonococcal urethral discharge. In 2009, however, reduced susceptibility to this antibiotic was observed with the proportion of isolates exhibiting resistance increasing from 3.8 % in 2009 to 79.5 % in 2018. The data presented here show that high-level resistance to ciprofloxacin was associated with distinct core genome groups that are globally prevalent, with one particular core genome group more frequently found in Africa. These data improve our understanding of *N. gonorrhoeae* originating from central Africa and enhance public health initiatives. It will be of interest for the ongoing surveillance of *N. gonorrhoeae* resistance to antibiotics and for development of the infrastructure and methodology needed to continue the surveillance of gonorrhoea in Africa.

## Introduction

Gonorrhoea, caused by the bacterium *Neisseria gonorrhoeae *, is a widespread global bacterial sexually transmitted infection (STI) with the World Health Organisation (WHO) estimating there were 82.4 million people newly infected with *N. gonorrhoeae* in 2020 [1]. A total of 11.4 million cases have been reported by the WHO in the African Region [2], but the burden of infection is likely to be higher, as gonorrhoea is poorly documented due to limited diagnostic capacity and/or reporting systems [3–5].

Effective antimicrobial treatment is crucial to avoid the severe complications of gonorrhoea [6]. In Cameroon, the recommended first-line treatment for both men and women is ciprofloxacin, with, in addition, doxycycline administered to treat *Chlamydia trachomatis* co-infections in men. In contrast, women will be offered metronidazole to treat potential trichomoniasis co-infections. Second-line treatment includes ceftriaxone and doxycycline in both men and women. However, antimicrobial resistance (AMR) has been increasingly observed globally towards ceftriaxone, ciprofloxacin and azithromycin [7]. For example, in Vietnam in 2019, 98.3% of studied strains (*n*=409) were resistant to ciprofloxacin [8], with the prevalence of gonococci resistant to ciprofloxacin remaining high in the USA [9]. A high prevalence of resistance to penicillin, tetracycline and ciprofloxacin has also been described in South Africa in 2018 [10] with similar patterns observed in Cameroon [11, 12].

Enhanced, quality-assured surveillance is necessary to detect emerging AMR patterns exhibited by gonococci and inform on STI treatment guidelines nationally, regionally and globally [13]. The WHO has developed a global action plan to control *N. gonorrhoeae* AMR and limit its impact by advocating better monitoring of resistant strains, including improved prevention, diagnosis and treatment that are tailored to the differing AMR patterns exhibited by gonococci globally [1]. This study investigated *N. gonorrhoeae* AMR in Yaoundé, Cameroon, from 2019 to 2020 to six antimicrobials as recommended by the European Committee on Antimicrobial Susceptibility Testing (EUCAST). We identify isolates with decreased susceptibility to several antimicrobial families. We characterize whole genome sequence (WGS) data belonging to these isolates and compare these with 1355 publicly available genomes, retrieved from PubMLST that belong to the same core genome lineages as those identified in the Cameroonian gonococci. In so doing, we identify core genome lineages circulating in Africa that are resistant to ciprofloxacin, tetracycline and penicillin. This study presents a genomic portrait of gonococci circulating in Africa. It describes how the *N. gonorrhoeae* core gene multilocus sequence typing (cgMLST) scheme can be used to find related genomes for comparative analyses.

## Methods

### Strains and antimicrobial susceptibility determination

From January 2019 to December 2020, clinical gonococcal isolates (one isolate per patient) were cultured at the University Hospital Center, Exact Laboratory (Yaoundé) and Prima Laboratory (Yaoundé). Quality control of the *N. gonorrhoeae* strains collected in these different health structures was carried out at the National Public Health Laboratory. Isolates, cultured from urethral swabs or high vaginal/cervical swabs, were obtained after informed consent from outpatients with urethral discharge (males *n*=4) or vaginal discharge (females *n*=2, one of whom was pregnant) attending hospitals in Yaoundé, Cameroon.

Patient socio-demographic data including age and sex were collected (Table 1). Isolates were cultured using chocolate agar supplemented with polyvitex. Species identification was done through Gram-staining, oxidase test and API NH test (bioMérieux). AMR testing was performed in Oxford using an E-test (bioMérieux) for ciprofloxacin, penicillin G, ceftriaxone, cefixime, azithromycin and tetracycline and the results were interpreted with reference to EUCAST standards (Table 1). Isolates were cultured on chocolate GC Selective Agar (Oxoid) at 37 °C in a 5 % CO_2_-enriched atmosphere for 18–24 h. The gonococcal WHO strains L and P were used as reference [14]. Clinical breakpoints were determined according to EUCAST clinical breakpoints v12. In brief, azithromycin isolates with a minimum inhibitory concentration (MIC) <0.5 mg l^−1^ were assigned as susceptible and MIC >1 mg l^−1^ resistant. For cefixime and ceftriaxone, isolates with MIC ≤0.125 mg l^−1^ were assigned as susceptible or as resistant in the case of MIC >0.125 mg l^−1^. For ciprofloxacin, isolates with MIC ≤0.03 were assigned as susceptible or as resistant with MIC >0.06 mg l^−1^. For penicillin, isolates with MIC ≤0.06 were assigned as susceptible or resistant with MIC >1 mg l^−1^, indicating that isolates falling within this range will have intermediate resistance. For tetracycline, isolates with MIC ≤0.5 were assigned as susceptible or resistant with MIC >0.5.

**Table 1.** Isolate characteristics and antimicrobial susceptibility phenotypes

Strain (PubMLST id)	Source and gender	Isolation date	AZM	CIP	CFM	CRO	PEN	TET
NG 31052019 (117540)	Urethral (M)	05/2019	0.094 (S)	>32 (R)	<0.002 (S)	0.004 (S)	2 (R)	32 (R)
NG 27082019 (117541)	Endocervical (F)	08/2019	0.064 (S)	1.5 (R)	<0.002 (S)	<0.002 (S)	0.047 (S)	8 (R)
NG 20042303 (117543)	Urethral (M)	04/2020	0.047 (S)	4 (R)	<0.002 (S)	0.02 (S)	12 (R)	12 (R)
NG 13112001 (117545)	Urethral (M)	11/2020	0.032 (S)	<0.002 (S)	<0.002 (S)	<0.002 (S)	16 (R)	8 (R)
NG 14112002 (117546)	Endocervical (F) PREGNANT	11/2020	0.016 (S)	2 (R)	<0.002 (S)	0.003 (S)	12 (R)	12 (R)
NG 14112003 (117547)	Urethral (M)	11/2020	0.064 (S)	0.5 (R)	<0.002 (S)	0.006 (S)	0.094 (I)	8 (R)

AZM, azithromycin; CFM, cefixime; CIP, ciprofloxacin; CRO, ceftriaxone; F, female; I, intermediate; M, male; PEN, penicillin; R, resistant; S, susceptible; TET, tetracycline.

### Whole genome sequencing and assembly annotation

*N. gonorrhoeae* was grown overnight on chocolate GC Selective Agar (Oxoid) in 5 % CO_2_ at 37 °C and genomic DNA was extracted using the Wizard Genomic DNA Purification Kit (Promega). Genomic DNA libraries were prepared using the Nextera XT Library Prep Kit (Illumina) following the manufacturer’s protocol. Libraries were then sequenced using the Illumina HiSeq sequencing platform and a 250 bp paired-end protocol (https://microbesng.com). Reads were adapter trimmed using Trimmomatic 0.30 with a sliding window quality cutoff of Q15 [15]. *De novo* assembly was performed using SPAdes version 3.7 with default settings [16].

The resulting assemblies were deposited in the PubMLST *Neisseria* database which uses the Bacterial Isolate Genome Sequence Database (BIGSdb) software [17]. BIGSdb includes ‘autotagger’ and ‘autodefiner’ tools, which scan deposited genomes against defined loci identifying alleles sharing ≥98 % sequence identity. This process runs in the background and automatically updates isolate records with specific allele numbers, marking regions on assembled contiguous sequences (contigs) for any of the defined loci. Loci with sequence identity <98 % are manually checked and curated. In PubMLST, locus sequence definitions have been established for over 2600 protein-encoding genes, annotated as loci with the NEIS prefix and the majority of these have been organized into schemes dependent on function, such as AMR. Once deposited in PubMLST, genomes were thus automatically annotated with all of the loci defined in the database including the following AMR-associated loci: NEIS0149 (*rpsE*), NEIS0414 (*ponA*), NEIS1320 (*gyrA*), NEIS1525 (*parC*), NEIS1609 (*folP*), NEIS1635 (*mtrR*), pro_NEIS1635 (*mtrR* promoter sequence), NEIS1753 (*penA*), NEIS2020 (*porB*) and 23S_rRNA. Alleles for each of these loci were downloaded for each isolate and using the molecular evolutionary analysis software mega v11 [18], deduced amino acid sequences were aligned and polymorphic sites associated with antimicrobial resistance were identified. These amino acid substitutions were subsequently annotated for each allele sequence. *N. gonorrhoeae* can possess two plasmids, the conjugative (pConj) and β-lactamase (p*bla*) plasmids, which confer high-level resistance against tetracycline and β-lactams, respectively. Loci from these plasmids have previously been defined in PubMLST and allow their presence to be detected [19]. In particular, NEIS2210 encodes the *tetM* gene and confers resistance to tetracycline, while NEIS2357 encodes the beta-lactamase gene *blaTEM* and confers high-level resistance to penicillin.

MLST and NG MAST v2.0 sequence types (STs) were manually assigned in PubMLST [20]. NG STAR indexes the variability found in nucleotide sequence fragments from seven genes associated with AMR (*penA, mtrR, porB, ponA, gyrA, parC* and 23S rRNA) and new alleles were submitted for curation to the online publicly available NG STAR database (https://ngstar. canada.ca) [21]. NG STAR clonal complexes (CCs) have previously been described where STs are grouped into a CC on the basis of these sharing five or more alleles with a central genotype [22]. NG STAR CCs were therefore implemented in PubMLST using published definitions and allowing NG STAR CCs to be identified.

### Phylogenetic analyses of gonococci belonging to cgc_300 groups 21, 41, 243 and 498

Over 1600 genes have been identified as ‘core’ in *N. gonorrhoeae* and these genes have been defined in the PubMLST database (https://pubmlst.org/neisseria) and form part of the *N. gonorrhoeae* cgMLST version 1.0 scheme [23]. cgSTs are assigned for each isolate and, through single-linkage clustering and the use of increasing allelic difference thresholds, isolates can be grouped into related core genome groups. Thus, using a threshold of 300 or fewer locus differences, where isolates dissimilar in 300 or fewer loci in the core genome form lineages of related gonococci, the Cameroonian gonococci were found to cluster into four core genome groups: Ng_cgc_300 : 21, 41, 243 and 498 (Table 2). Additional gonococci belonging to Ng_cgc_300 : 21, 41, 243 and 498 core genome groups were searched for in the PubMLST isolate database using the ‘Allele designations/scheme fields’ function located on the Search or browse page. This identified: 571 gonococci belonging to Ng_cgc_300 : 21, 488 to Ng_cgc_300 : 41, 245 to Ng_cgc_300 : 243 and 51 to Ng_cgc_300 : 498 (Table S1). This resulted in 1355 gonococci, the genomes of which were compared using the *N. gonorrhoeae* cgMLST v1.0 scheme [23]. A neighbour-joining phylogenetic tree was generated from aligned, concatenated nucleotide sequences from the *N. gonorrhoeae* cgMLST using the iTOL plugin tool on PubMLST [24]. The tree was then annotated by core genome group, plasmid presence and occurrence of amino acid substitutions conferring resistance to ciprofloxacin in GyrA and ParC. A minimum spanning tree (MST) was also generated using the GrapeTree plugin on PubMLST [25]. This tool compares allelic profiles, clustering gonococci sharing alleles across the core genome. GrapeTree was run by selecting the *N. gonorrhoeae* cgMLST v1.0 scheme and including the Ng_cgc_200 and 300 groups as metadata for MST annotation.

**Table 2.** Isolate strain designations and antimicrobial resistance (AMR) genotypes found

Strain (PubMLST id)	Accession (Biosample)	MLST	NG MAST v2.0	NG STAR (CC)	Core genome group (Ng_cgc_300)	CIP NEIS1320 (*gyrA*) and NEIS1525 (*parC*)	AZM 23S rRNA and NEIS0149 (*rpsA*)	TET NEIS2210 (*tetM*)	BETA-LACTAMS NEIS0414 (*ponA*) NEIS1635 (*mtrR*) proNEIS1635 (*mtrR* promoter) NEIS1753 (*penA*) NEIS2020 (*porB*) NEIS2357 (*blaTEM*)
NG 31052019 (117540)	ERS14364953 (SAMEA112254100)	1588	20 972	567 (CC309)	21	NEIS1320 allele 234 (S91F; D95G) NEIS1525 allele 411 (S87N)	23S rRNA allele 231 (WT) NEIS0149 allele 3 (WT)	NEIS2210 allele 2	NEIS1753 allele 228 (F504L, non-mosaic, XIX) NEIS0414 allele 13 (L421P) NEIS2020 allele 4491 (PIB G120K; A121G) NEIS2357 allele 3 NEIS1635 allele 368 (internal stop codon) proNEIS1635 allele 6 (WT)
NG 27082019 (117541)	ERS14364954 (SAMEA112254101)	13 781	3800	4860 (CC1054)	498	NEIS1320-14 (S91F; D95G) NEIS1525-261 (WT)	23S rRNA allele 521 (WT) NEIS0149 allele 3 (WT)	NEIS2210 allele 2	NEIS1753 allele 21 (F504L and P551S, non-mosaic, XII) NEIS0414 allele 13 (L421P) NEIS2020 allele 966 (PIB, WT) NEIS1635 allele 18 (WT) proNEIS1635 allele 6 (WT)
NG 20042303 (117543)	ERS14364955 (SAMEA112254102)	7363	1856	4931 (CC-)	41	NEIS1320-234 (S91F; D95G) NEIS1525-2089 (S87N)	23S rRNA allele 231 (WT) NEIS0149 allele 3 (WT)	NEIS2210 allele 2	NEIS1753 allele 294 (F504L, non-mosaic, XIV) NEIS0414 allele 13 (L421P) NEIS2020 allele 4490 (PIA) NEIS2357 allele 3 NEIS1635 allele 875 (WT) proNEIS1635 allele 6 (WT)
NG 14112003 (117547)	ERS14364956 (SAMEA112254103)	1588	20 975	3318 (CC-)	21	NEIS1320-234 (S91F; D95G) NEIS1525-246 (S87N)	23S rRNA allele 231 (WT) NEIS0149 allele 3 (WT)	NEIS2210 allele 2	NEIS1753 allele 294 (F504L, non-mosaic, XIV) NEIS0414 allele 13 (L421P) NEIS2020 allele 4494 (PIA) NEIS1635 allele 16 (WT) proNEIS1635 allele 6 (WT)
NG 14112002 (117546)	ERS14364957 (SAMEA112254104)	15 667	20 974	4861 (CC1054)	498	NEIS1320-14 (S91F; D95G) NEIS1525-630 (WT)	23S rRNA allele 521 (WT) NEIS0149 allele 3 (WT)	NEIS2210 allele 2	NEIS1753 allele 285 (F504L and P551S, non-mosaic, IX) NEIS0414 allele 223 (WT) NEIS2020 allele 4493 (PIB, WT) NEIS2357 allele 3 NEIS1635 allele 18 (WT) proNEIS1635 allele 6 (WT)
NG 13112001 (117545)	ERS14364958 (SAMEA112254105)	1599	20 973	463 (CC352)	243	NEIS1320-19 (WT) NEIS1525-161 (WT)	23S rRNA allele 495 (WT) NEIS0149 allele 3 (WT)	NEIS2210 allele 2	NEIS1753 allele 294 (F504L, non-mosaic, XIV) NEIS0414 allele 8 (WT) NEIS2020 allele 4492 (PIB, WT) NEIS2357 allele 3 NEIS1635 allele 18 (WT) proNEIS1635 allele 6 (WT)

AZM, azithromycin; CIP, ciprofloxacin; TET, tetracycline; beta-lactams, penicillin, ceftriaxone and cefixime. Alleles identified are depicted along with mutations.

One Cameroonian isolate was found to be MLST ST-7363. Gonococci belonging to this ST are known to form two clusters when analysing the core genome that correspond to the presence or absence of mosaic *penA* alleles, which are known to confer resistance to third-generation cephalosporins [26] As a result, the genome from this isolate was compared with other gonococci with this same ST for which 895 records were retrieved from PubMLST (Table S2). Since some ST-7363 isolates are known to possess mosaic *penA* alleles and be highly antimicrobial resistant – for example the highly resistant isolate H041 (WHO-X) is ST-7363 [27] – an MST was generated comparing the *N. gonorrhoeae* cgMLST in the 895 ST-7363 gonococci. The resulting tree was then annotated by core genome group at the 300 or fewer locus difference threshold and type of mosaic *penA* allele found if any.

## Results

### Antimicrobial susceptibility phenotypes in the Cameroonian gonococci

Six patients were recruited in this study (Table 1). Among these, four were male and two were female. Patient ages ranged from 18 to 40 years. Six *N. gonorrhoeae* isolates were obtained from these patients dating from 2019 (*n*=2) and 2020 (*n*=4) (Table 1).

**Fig. 1.**
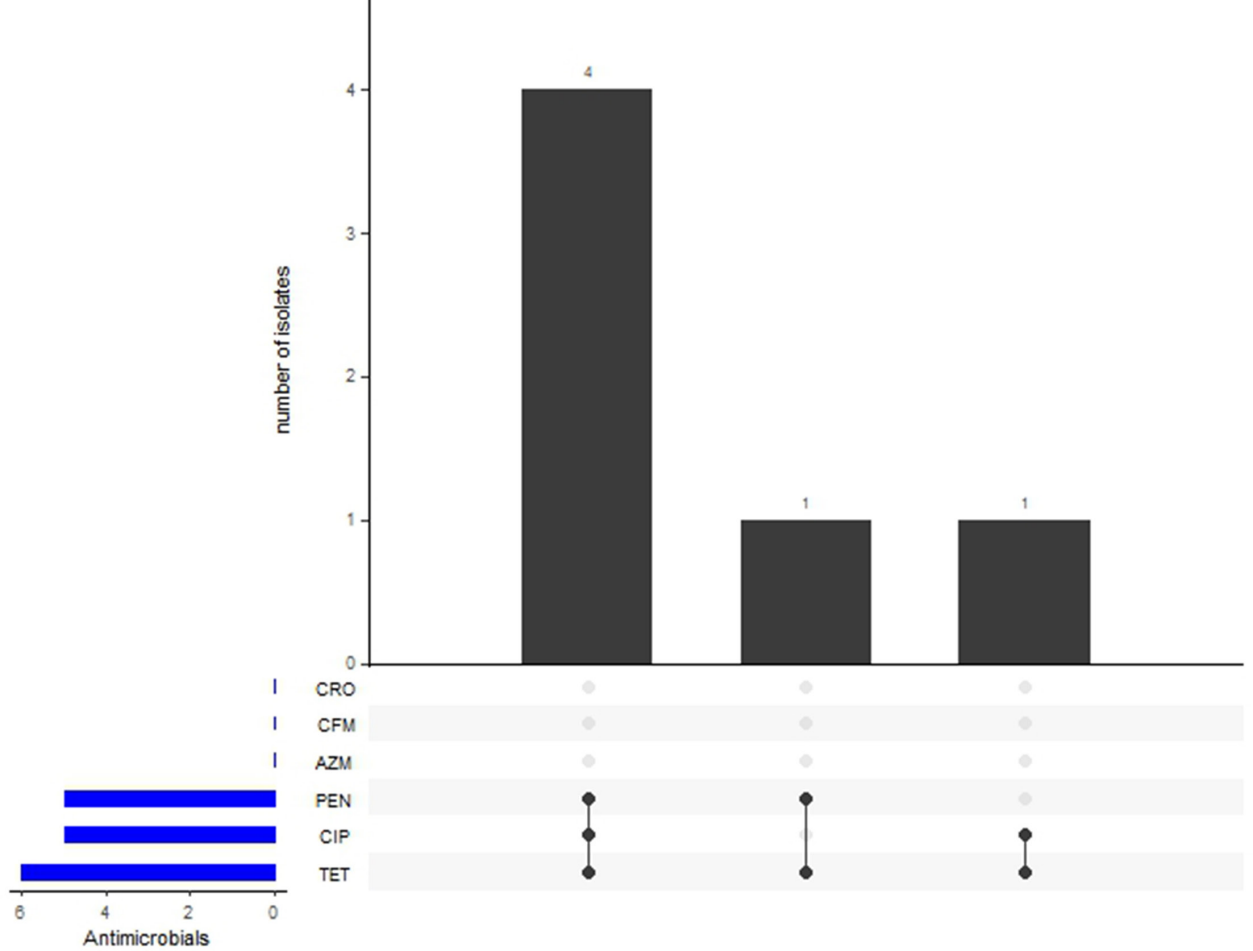
UpSet plot depicting AMR phenotype associations in Cameroonian *N. gonorrhoeae* isolates. Observed concomitant resistant phenotypes exhibited by the Cameroonian isolates. This UpSet plot shows the different sets of resistances observed across the dataset. The top bar plot indicates how many isolates were resistant to each specific group of antibiotics. The corresponding set of antibiotics is indicated below the top bar by black dots. The bar plot to the left shows how frequently resistance to each antibiotic occurred regardless of which set of resistances it appeared within. The UpSet plot was generated using the UpSetR package in R v4.2.3 [40]. CRO: ceftriaxone, CFM: cefixime, AZM: azithromycin, PEN: Penicillin, CIP: ciprofloxacin, TET: tetracycline

Four strains were resistant to penicillin with MIC ranges from 2 to 16 mg l^−1^, six were resistant to tetracycline with MIC ranges from 8 to 32 mg l^−1^ and five were resistant to ciprofloxacin with MIC ranges from 0.5 to >32 mg l^−1^. One isolate had intermediate resistance to penicillin (MIC 0.094 mg l^−1^). None of the isolates were resistant to azithromycin (MIC ranges from 0.016 to 0.094 mg l^−1^) and none exhibited reduced susceptibility to cefixime or ceftriaxone with the highest MIC detected being 0.02 mg l^−1^ (Fig. 1).

### Genomic characterization of AMR determinants in the Cameroonian gonococci

#### Macrolide resistance

Macrolides such as azithromycin bind to the 50S ribosomal unit and inhibit protein synthesis. Nucleotide polymorphisms that include C2611T and A2095G in 23S rRNA (* Escherichia coli* numbering, this corresponds to C2599T and A2143G in the gonococcus) result in reduced binding affinity of 23S with the macrolide [28]. The Cameroonian gonococci sequenced in this study possessed 23S rRNA alleles 231, 495 and 521, which do not contain such nucleotide polymorphisms (Table 2). This was consistent with obtained MIC values in that all of the sequenced isolates were susceptible to this antibiotic (Table 1).

#### Fluoroquinolone resistance

Fluoroquinolones inhibit DNA metabolism by acting upon the DNA gyrase gene, *gyrA* (NEIS1320), and the topoisomerase IV gene, *parC* (NEIS1525). Amino acid substitutions S91F, D95N, D95G or D95A in GyrA decrease fluoroquinolone binding with these mutations alone providing low to intermediate resistance [29]. Additional mutations in ParC (NEIS1525) that include D86N, S87P and E91K induce higher resistance to fluoroquinolones [30]. These genes are designated as NEIS1320 (*gyrA*) and NEIS1525 (*parC*) in PubMLST. Of the five ciprofloxacin-resistant isolates, the amino acid substitutions S91F and/or D95G/D95A in GyrA and S87N in ParC were detected (Table 2) with the ciprofloxacin-susceptible isolate lacking these, consistent with obtained MIC values.

#### Resistance to beta-lactams including third-generation cephalosporins

Chromosomally mediated resistance to beta-lactams including penicillin and third generation cephalosporins such as cefixime and ceftriaxone is often the cumulative result of mutations in several genetic determinants including mosaic *penA* genes, non-synonymous mutations in the gene *ponA* which encodes the penicillin-binding protein 1, mutations in the *mtrR* repressor gene and/or its promotor sequence, and mutations in loop 3 of the outer membrane protein PorB variant PIB [31]. A total of four distinct NEIS1753 (*penA*) alleles were identified, none of which were mosaic. Of these, two contained the amino acid substitution F504L (NEIS1753 allele 228, type XIX non-mosaic NG STAR *penA* allele 19.001 and NEIS1753 allele 294, type XIV non-mosaic NG STAR *penA* allele 14.001). The remaining two alleles, NEIS1753 allele 21 (type XII non-mosaic NG STAR *penA* allele 12.001) and NEIS1753 allele 285 (type IX NG STAR *penA* allele 9.001) contained the mutations F504L and P551S (Table 2). Four of the isolates possessed NEIS0414 (*ponA*) allele 13 which contains the amino acid mutation L421P, with the remaining two isolates possessing wild-type NEIS0414 alleles 8 and 223 (Table 2). One isolate possessed an *mtrR* gene with a premature stop codon (NEIS1635-368), but none of the isolates possessed mutations in the promoter sequence. Two isolates possessed PIA PorB variants (isolates NG 20042303 and NG 14112003) with the remaining four isolates harbouring PIB PorB variants. Only one PorB allele 4491 (isolate NG 31052019) contained the amino acid substitutions G120K and A121G associated with AMR (Table 2). These genotypic results are consistent with MIC phenotypes in that none of the sequenced isolates were resistant to third-generation cephalosporins (Table 1). The high MIC values obtained towards penicillin and tetracycline were found to be plasmid-mediated with the intermediate MIC value (0.094 mg l^−1^) exhibited by isolate NG 14112003 a result of mutations in NEIS0414 (*ponA*) and NEIS1753 (*penA*).

#### Plasmid-mediated resistance

All six gonococci possessed pConj plasmids containing the *tetM* gene (NEIS2210) conferring resistance to tetracycline. Of these, four also possessed beta-lactamase plasmids. This was consistent with the MIC values identified towards tetracycline and penicillin in that all six gonococci were resistant to tetracycline with the four isolates possessing beta-lactamase plasmids resistant to penicillin (Table 2 and Fig. 1).

#### MLST, NG MAST and NG STAR profiles

Five different MLST STs were identified: ST-1588, ST-1599, ST-7363, ST-13781 and ST-15667. ST-1588, ST-1599 and ST-7363 are globally distributed, first defined in 2001 (ST-1588 and 1599) and 1998 (ST-7363). There were only three other ST-13781 isolates in PubMLST, two identified in the UK in 2013 with another found in South Africa. ST-15667 has only been identified in Africa (Malawi and Cameroon). Some ST-7363 gonococci have been associated with AMR and, as a result, the ST-7363 Cameroonian isolate was compared with an additional 895 gonococci with this ST (Table S2). An MST was generated revealing that, although these isolates possess the same MLST ST, these had distinct core genome ancestries (Fig. S1A). Mosaic *penA* alleles were predominantly associated with MLST ST-7363 gonococci belonging to Ng_cgc_300 : 8 (Fig. S1B).

All of the isolates had distinct NG MAST profiles, four of which were newly identified in this study. Similarly, each isolate had unique NG STAR STs (Table 2). Of the NG STAR STs identified, ST-567 (CC309) was the most prevalent in PubMLST, found in 35 other isolates, the majority of which were MLST ST-1588 and belonged to Ng_cgc_300 : 21 (Table S1). Of these, 23/29 (79 %) possessed pConj plasmids with 14 of these also containing p*bla* plasmids. The remaining NG STAR profiles were not widely found with four of the six isolates possessing NG STAR STs unique to Cameroon.

#### Genomic analyses of isolates belonging to Ng_cgc_300 groups 21, 41, 243 and 498

**Fig. 2.**
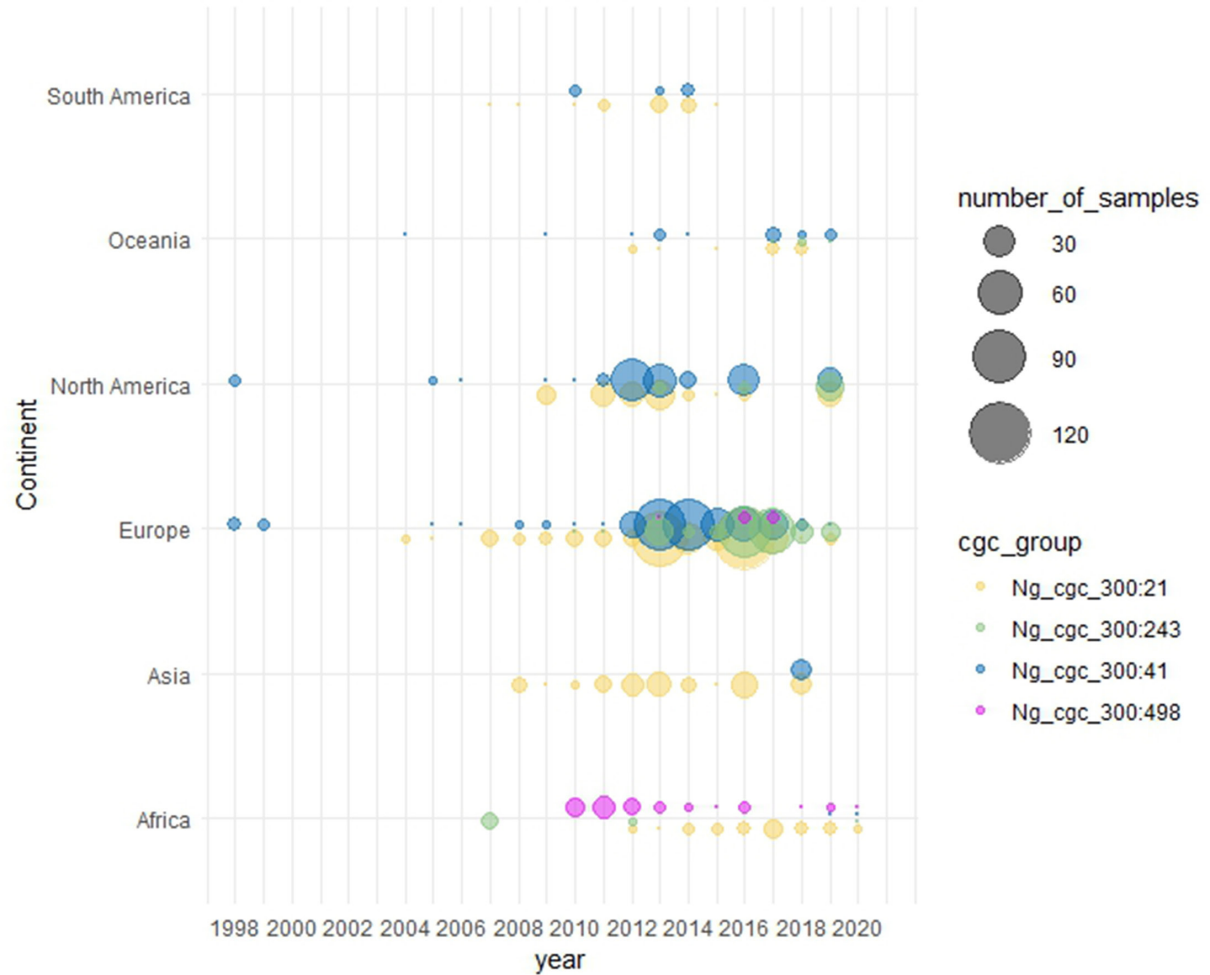
Temporal and geographical distribution of cgc_groups_300 : 21, 41, 243 and 498 in PubMLST. Searches of PubMLST for gonococci belonging to the core genome groups identified in the Cameroonian isolates identified: 571 gonococci belonging to Ng_cgc_300 21, 488 to Ng_cgc_300 41, 245 to Ng_cgc_300 243 and 51 to Ng_cgc_300 498. Temporal and geographical distributions were plotted using ggplotR2 package 3.4.1 [41] revealing that many of the gonococci belonging to Ng_cgc_300 : 498 originate from Africa.

Using a 300 allelic difference threshold, the Cameroonian gonococci belonged to four core genome groups: Ng_cgc_300 : 21, 41, 243 or 498. Each core genome group comprises related gonococci that have 300 or fewer locus differences in the core genome. Allelic differences occurring in more than 300 loci will be present between core genome groups. Searches of PubMLST identified 571 other gonococci belonging to Ng_cgc_300 : 21, with 488 gonococci belonging to Ng_cgc_300 : 41. In both core genome groups, isolates were globally distributed and dated from 2004 to 2020 (Ng_cgc_300 : 21) and from 1998 to 2019 (Ng_cgc_300 : 41) (Table S1 and Fig. 2). Searches for Ng_cgc_300 : 243 identified 245 gonococci belonging to this core genome group (Table S1). These dated from 2007 to 2020 and originated from Africa, Europe, North America and Oceania. A total of 51 gonococci belonged to Ng_cgc_300 : 498. These dated from 2010 to 2019, the majority originating from Africa (*n*=44) with the remaining seven isolates from Europe (Table S1 and Fig. 2).

**Fig. 3.**
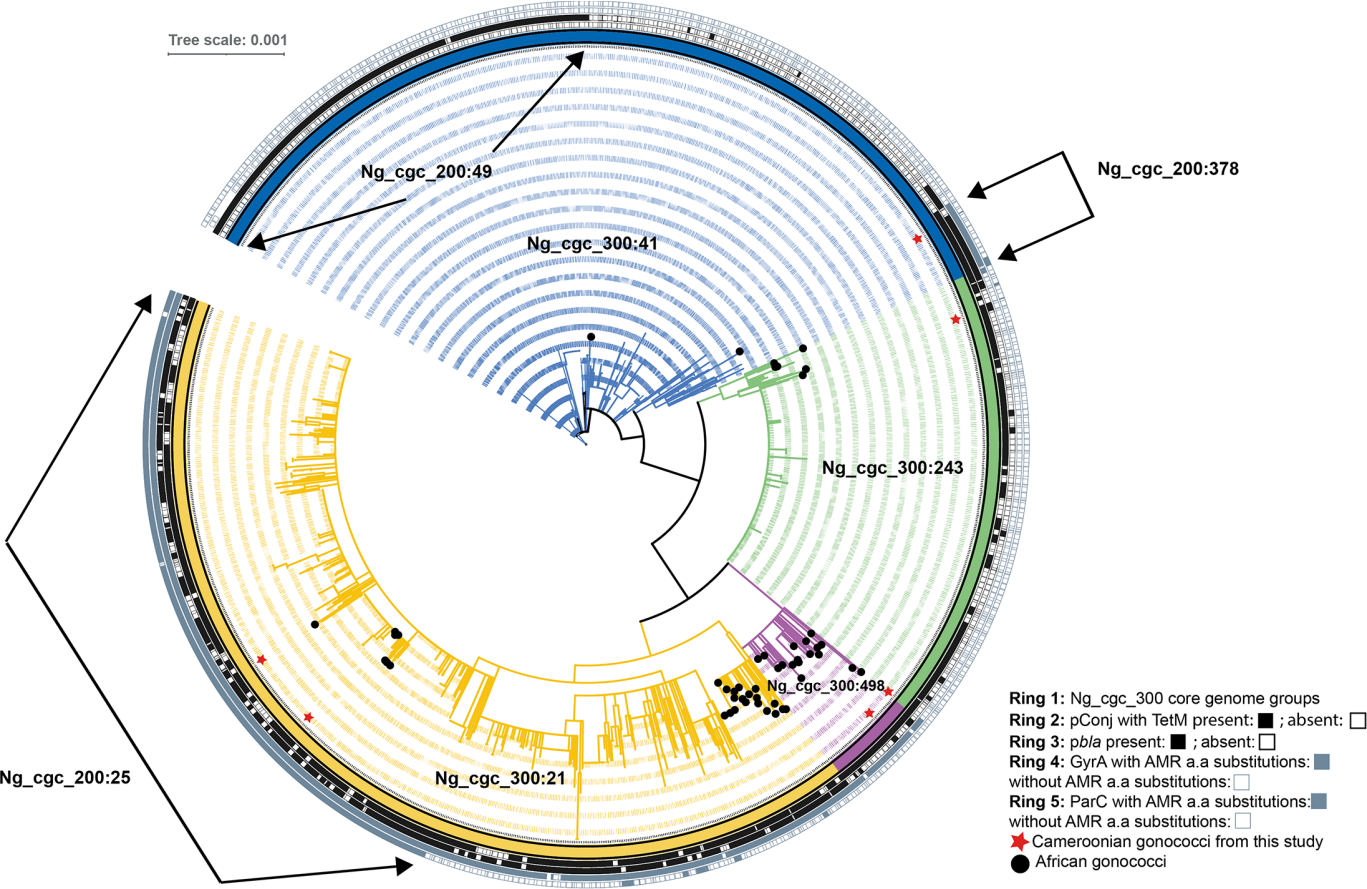
Phylogenetic analyses of 1355 *N *. *gonorrhoeae* belonging to Ng_cgc_300 core genome groups 21, 41, 243 and 498. These isolates collectively date from 1998 to 2020 and originate from six continents. The neighbour-joining tree was generated through the concatenated alignment of the *N. gonorrhoeae* cgMLST v1.0 core genome and annotated using iTOL [24]. The inner coloured strip indicates core genome groups with Ng_cgc_300 : 21 depicted in yellow, Ng_cgc_300 : 41 in blue, Ng_cgc_300 : 243 in green and Ng_cgc_300 : 498 in purple. The next two rings represent pConj and p*bla* plasmid occurrence respectively with filled black squares indicative of plasmid presence and open black squares plasmid absence. Filled grey squares denote the presence of amino acid substitutions known to confer resistance to ciprofloxacin in GyrA and/or ParC with open grey squares indicating the absence of these substitutions. Red stars denote the Cameroonian gonococci sequenced in this study. Black circles denote African gonococci. A sub-lineage within Ng_cgc_300 : 21 can be observed consisting of isolates possessing both pConj and p*bla* plasmids as well as *gyrA* and *parC* alleles containing amino acid substitutions known to confer high-level resistance to fluoroquinolones. Using a 200 or fewer locus difference threshold, this sub-lineage consists of 325 gonococci belonging to Ng_cgc_200 : 25. These isolates originate from all six continents and date from 2005 to 2019.

A neighbour-joining phylogenetic tree was generated from the 1355 gonococci belonging to the core genome groups identified in this study using the concatenated nucleotide sequence alignment of the *N. gonorrhoeae* cgMLST v1.0 core genome. This was then annotated by core genome group, plasmid occurrence and presence of amino acid substitutions in GyrA (NEIS1320) and/or ParC (NEIS1525) (Fig. 3). Distinct clusters were apparent that were congruent with the core genome groups at the 300 or fewer locus threshold. These clusters were identical to those obtained using MSTs (Fig. S2A) which clusters isolates based on allelic profiles rather than nucleotide sequence data.

The pConj plasmid was prevalent in Ng_cgc_300 groups: 21 (455/571, 80 %), 243 (189/245, 77 %) and 498 (51/51, 100 %) but was absent in the majority of Ng_cgc_300 : 41 isolates (40/488, 8 %). Within core genome group Ng_cgc_300 : 41, the pConj plasmid was only found in a sub-lineage consisting of 36 isolates, including the Cameroonian isolate NG 20042303 (id 117543), which possessed both pConj and p*bla* plasmids, and *gyrA* (NEIS1320) allele 234 with the AMR-associated amino acid substitutions S91F and D95G (Fig. 3). This sub-lineage comprised isolates dating from 2010 to 2020 that originated from Asia, Africa, Europe and South America with the Cameroonian isolate the sole African gonococcus. A total of 24/36 (67 %) of these belonged to Ng_cgc_200 : 378, using the more stringent 200 or fewer locus difference threshold, indicative of a globally distributed group of closely related gonococci (Figs 3 and S2B). The remainder of isolates belonging to Ng_cgc_300 : 41 possessed wild-type AMR genes predicted to be susceptible to tetracycline, penicillin and ciprofloxacin. The p*bla* plasmid was most frequently found in Ng_cgc_300 : 21 (411/571, 72%) with a sporadic distribution in Ng_cgc_300 : 243 and Ng_cgc_300 : 498. Two clusters of isolates possessing p*bla* were, however, apparent in Ng_cgc_300 : 41, including lineage Ng_cgc_200 : 378 and an additional larger cluster of isolates belonging to Ng_cgc_200 : 49, consisting of 185 isolates. This was a clonal group with 150/185 (81 %) MLST ST-1584, 69/184 (37 %) NG STAR ST-178 (CC178) and 119/185 (64 %) NG MAST ST-26.

The distribution of isolates possessing *gyrA* (NEIS1320) and/or *parC* (NEIS1525) alleles containing amino acid substitutions known to confer resistance to fluoroquinolones varied by core genome group. For example, the majority of isolates belonging to Ng_cgc_300 : 21 (553/571, 97%) possessed GyrA alleles (NEIS1320) with amino substitutions known to confer resistance with 548/553 (99 %) of alleles with the double mutations, S91F/D95A and 5/553 (1 %) with the mutations S91F/D95G. A total of 43/51 (84 %) Ng_cgc_300 : 498 isolates possessed GyrA (NEIS1320) alleles with amino acid substitutions conferring resistance and with 42/43 (99 %) harbouring the double mutations S91F/D95G. A sub-lineage within Ng_cgc_300 : 21 was observed consisting of isolates possessing both pConj and p*bla* plasmids as well as GyrA (NEIS1320) and ParC (NEIS1525) alleles, with the amino acid substitutions S91F/D95A (GyrA) and S87N (ParC) conferring high-level resistance to fluoroquinolones. Using a 200 or fewer locus difference threshold, this sub-lineage consisted of 325 gonococci belonging to Ng_cgc_200 : 25 (Figs 3 and S2B) and included isolates originating from all six continents, dating from 2005 to 2019. A total of 320/325 (98 %) of these isolates also possessed PonA with the L421P amino acid substitution and, of these, three originating from Thailand possessed mosaic *penA* allele 34 which has been associated with resistance to the third-generation cephalosporin cefixime [32]. Two of the Cameroonian isolates belonged to this sub-lineage although these did not have mosaic *penA* alleles.

## Discussion

This study investigated AMR phenotypes and genotypes exhibited by gonococci isolated in Yaoundé, Cameroon, from 2019 to 2020. A total of six gonococci were investigated and, of these, four exhibited resistant phenotypes to penicillin, tetracycline and ciprofloxacin, which is concerning given the many other gonococci circulating in Cameroon, Central and Western Africa for which we have limited genetic information. The results presented here revealed resistance to several antibiotics and included isolates with amino acid substitutions in both GyrA (S91F; D95A/G) and ParC (S87N) resulting in high-level resistance to ciprofloxacin, PonA alleles with the L412P amino acid mutation, and presence of both pConj and p*bla* plasmids (Tables 2 and S1). None harboured mosaic *penA* alleles, however, and only one isolate possessed a PorB PIB variant with the amino acid substitutions, G120K and A121G, known to confer resistance (Table 2). These data are consistent with the AMR phenotypic patterns identified previously in Cameroon where gonococci were resistant to penicillin, tetracycline and ciprofloxacin [11]. Similar trends have been observed in other African settings indicative of an increasing prevalence in resistance to these antibiotics across Africa [10, 19, 33–37].

Four of the six Cameroonian isolates possessed novel NG MAST profiles identified here (NG MAST STs: 20972, 20973, 20 974 and 20975) with isolates also possessing NG STAR STs that are infrequently found elsewhere (Table 2). For example, when querying PubMLST, there were 35 *N. gonorrhoeae* records NG STAR ST-567, eight records NG STAR ST-463 and two records NG STAR ST-3318. However, one isolate was MLST ST-7363 with some gonococci from this ST known to possess mosaic *penA* allele 10 associated with reduced susceptibility to third-generation cephalosporins [26]. The ST-7363 Cameroonian isolate found here harboured a non-mosaic *penA* allele and belonged to a distinct group of ST-7363 gonococci with a divergent core genome ancestry, indicative of horizontal gene transfer of MLST loci, precluding the use of these genes for the robust investigation of gonococcal population structure and as a means of identifying antimicrobial-resistant gonococci (Table S2 and Fig. S1). One group of ST-7363 isolates belonging to core genome group Ng_cgc_300 : 8, using the 300 or fewer locus threshold, contained most of the mosaic *penA* alleles (190/258, 74 %) and although the majority of these possessed mosaic *penA* allele 10 (184/258, 71 %), four other types were identified suggesting horizontal gene transfer and/or *de novo* mutations (*penA* alleles 34, 64, 75 and 147; Table S1 and Fig. S1) [23, 26].

Comparison of genomes using the *N. gonorrhoeae* cgMLST scheme increases the resolution needed to characterize the gonococcal population structure [23]. Searches of the PubMLST database retrieved genome sequences from gonococci belonging to the same cgMLST lineages as found in the Cameroonian isolates. This allowed these isolates to be examined in the context of existing data and compared with related gonococci. In so doing, a particular lineage was identified, Ng_cgc300 : 498, which predominantly comprised gonococci originating from Africa including two of the Cameroonian isolates investigated here (Figs 2 and 3). This lineage consisted of related gonococci that had on average 259 locus differences in the core genome. They possessed 18 different NG STAR STs, all of which belonged to NG STAR CC1054 [22] which is infrequently described and is not detected in high-income settings according to the latest Euro-GASP study [38], suggesting a lineage that is prevalent in Africa and rarely found elsewhere. Further associations between the core genome and AMR were found with, for example, a large sub-lineage within core genome group Ng_cgc_300 : 21, in which two of the Cameroonian isolates belonged, possessing GyrA alleles with S91F and D95G or D95A mutations and ParC alleles with S87R or S87N mutations as well as both plasmids (Fig. 3). Isolates in this sub-lineage dated from 2005 to 2019, indicating that amino acid mutations in GyrA and ParC associated with resistance to ciprofloxacin are maintained. The majority of isolates in this sub-lineage possessed the same *gyrA* (NEIS1320 allele 234, 303/325, 93 %), *parC* (NEIS1525 allele 246, 150/325, 46 %), *penA* (NEIS1753 allele 228, 294/325, 84 %) and *ponA* (NEIS0414, allele 13, 308/325, 95 %) alleles, with this allelic distribution remaining constant over time and indicating positive selection and a non-random association of these AMR alleles.

Previous studies have delineated the gonococcal population into two major lineages, A and B, with the latter found to consist of gonococci that were mainly susceptible and in which many of the African isolates included in that study belonged [39]. In contrast, the majority of the African isolates included here belonged to lineage A and were resistant to several antibiotics (Table S1). The exceptions were isolates from core genome groups Ng_cgc_300 : 41 and 243, which comprised gonococci (predominantly from Europe and North America) corresponding to lineage B. Accordingly, many of the isolates in these lineages were predicted to be susceptible to several antibiotics. A sub-lineage of Ng_cgc_300 : 41 was, however, observed that contained isolates predicted to be resistant to penicillin, tetracycline and ciprofloxacin indicative of a lineage that is adapting in response to changes in antibiotic consumption. Such adaptive changes may spread through a lineage, contributing to the emergence of AMR and it will therefore be important to monitor such AMR trends across gonococcal populations, both susceptible and resistant.

This study introduces key aspects in gonococcal genomics that can be used to enhance the surveillance of gonorrhoea in Cameroon and elsewhere. Although few Cameroonian isolates were investigated, their value has been maximized through the use of publicly available resources, to retrieve additional WGS data belonging to related gonococci identified using the *N. gonorrhoeae* cgMLST scheme. In so doing, we identify lineages that are circulating in Africa and elsewhere that are resistant to multiple antibiotics, which would not have been observed had we discarded this dataset due to its small size. It shows how no isolate collection is too small and that, particularly in resource-limited environments where obtaining viable isolates can be challenging, every isolate counts.

## Supplementary Data

Supplementary material 1

Supplementary material 2

Supplementary material 3
