# Supplementary material for: Phenotypic and genotypic characterization of Neisseria gonorrhoeae isolates from Yaoundé, Cameroon, 2019 to 2020
Source: Microb Genom. 2023 Aug 17;9(8):mgen001091. doi: 10.1099/mgen.0.001091 (PMC10483411; doi:10.1099/mgen.0.001091)

Supplementary Tables and figures

Supplementary Table 1. List of *N. gonorrhoeae* isolates retrieved from PubMLST and belonging to the core genome groups identified in this study.

Supplementary Table 2. Description of 895 ST-7363 *N. gonorrhoeae* isolates retrieved from PubMLST.

Supplementary Figure 1. Minimum spanning tree comparisons of ST-7363 gonococci. The MSTs are annotated using the 300 locus difference thresholds. Panel A MST annotated using the 300 or fewer locus difference threshold. Panel B annotated by mosaic *penA* presence. Each circle represents one isolate that is subsequently labelled by its corresponding core genome group (Panel A) or mosaic *penA* presence (Panel B). Numbers in brackets depict numbers of isolates belonging to these groups.

Supplementary Figure 2. Minimum spanning tree depicting cgMLST comparisons of gonococci belonging to the core genome groups identified in this study. The MSTs are annotated using the 200 and 300 locus difference thresholds. Panel A MST annotated using the 300 or fewer locus difference threshold. Panel B annotated using the 200 or fewer locus difference threshold. The sub-lineages 25 and 378 identified at the 200 or fewer locus difference threshold can be observed. Each circle represents one isolate that is subsequently labelled by its corresponding core genome group. Numbers in brackets depict numbers of isolates belonging to these groups.

Supplementary Figure 1. Minimum spanning tree comparisons of ST-7363 gonococci

Panel A

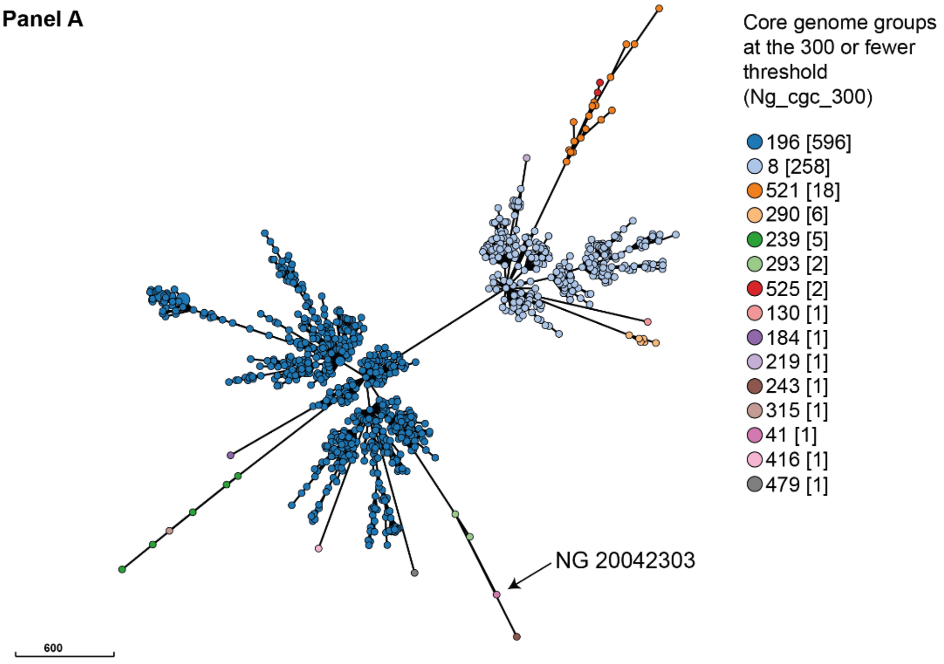

Panel B

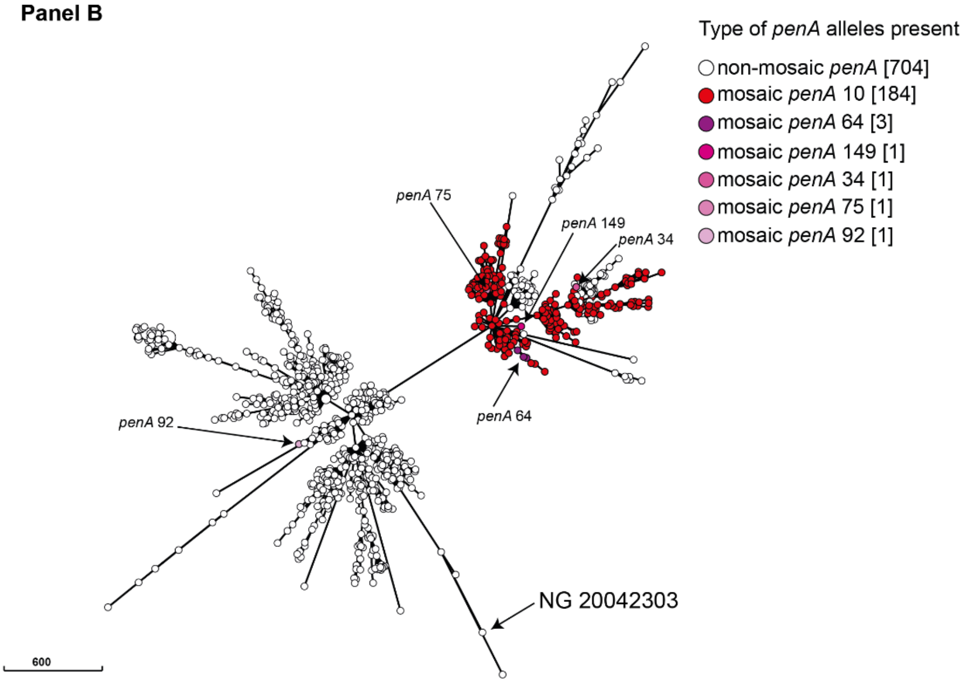

Supplementary Figure 2. Minimum spanning tree depicting *N. gonorrhoeae* cgMLST comparisons of gonococci belonging to the core genome groups identified in this study.

Panel A

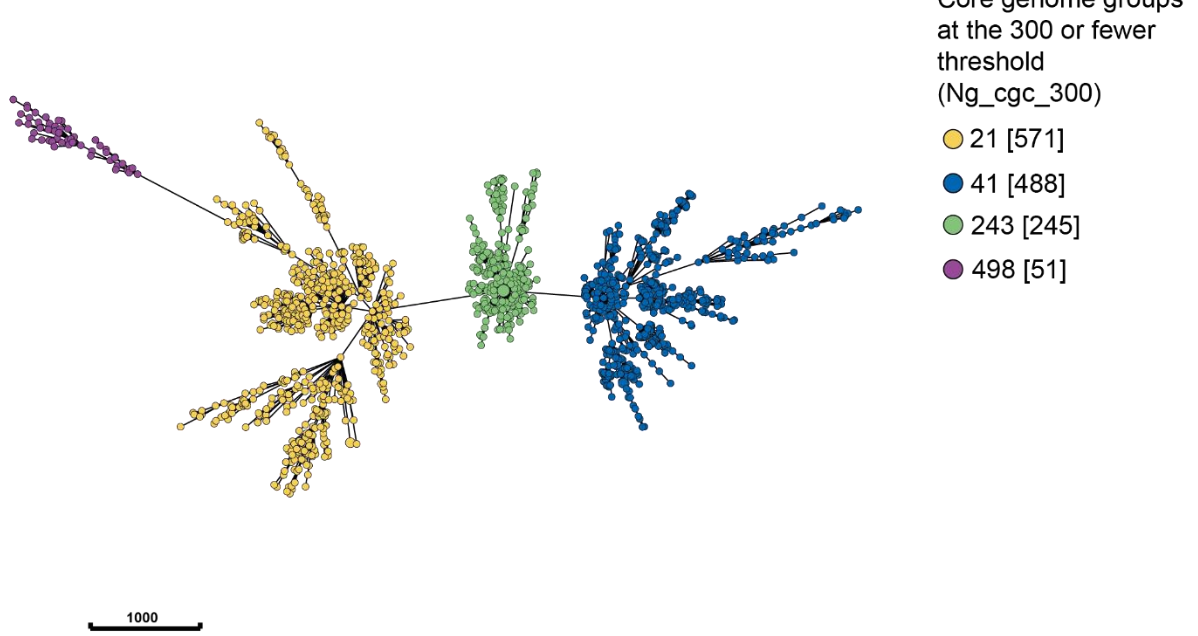

Panel B

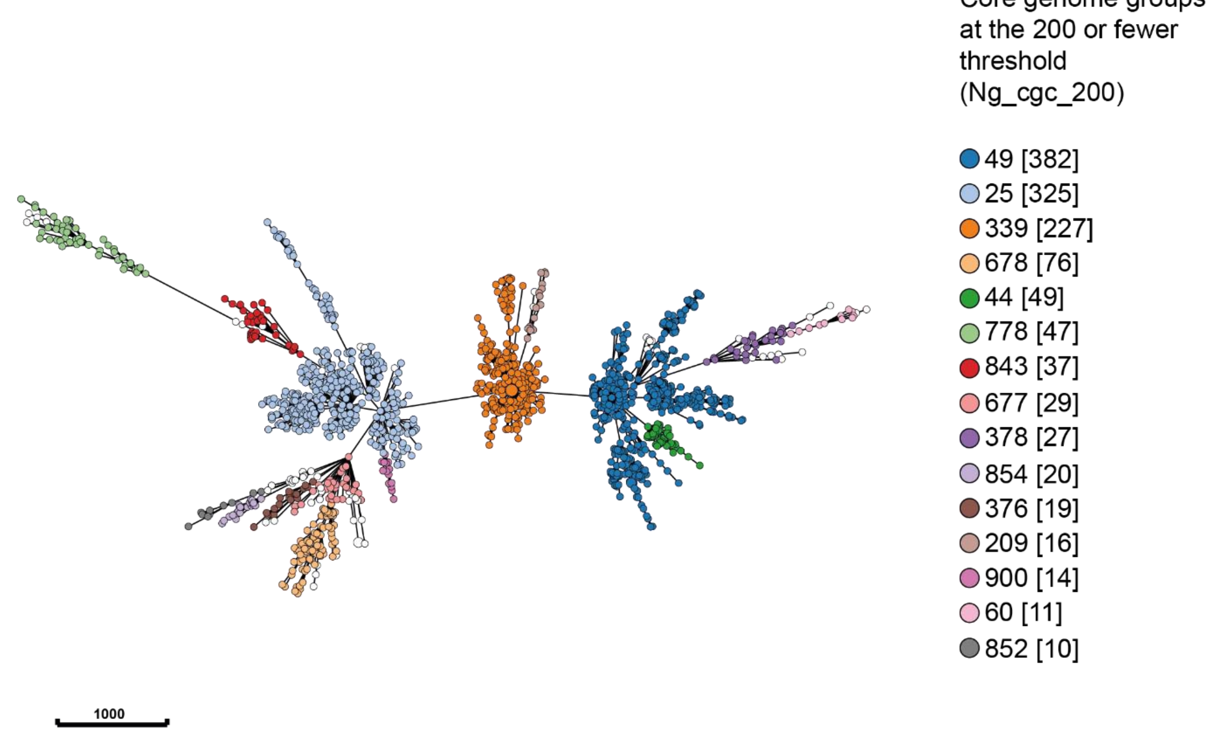

Supplement: Supplementary material 1 [file mgen-9-1091-s001.pdf]
